# Comparative Analysis Reveals the Adaptive Evolution of Olfactory System in the Swamp Eel ( *Monopterus albus* ) Based on the Chromosome‐Level Genome

**DOI:** 10.1002/ece3.73586

**Published:** 2026-04-29

**Authors:** Chuang Zhou, Zhongyi Wang, Chenhao Zhang, Zhaobin Song

**Affiliations:** ^1^ College of Life Sciences Sichuan Normal University Chengdu China; ^2^ Key Laboratory of Bio‐Resources and Eco‐Environment of Ministry of Education, College of Life Sciences Sichuan University Chengdu China; ^3^ Observation and Research Station of Sichuan Province of Fish Resources and Environment in Upper Reaches of the Yangtze River, College of Life Sciences Sichuan University Chengdu China

**Keywords:** adaptive evolution, chromosome‐level genome, comparative analysis, olfactory system, swamp eel

## Abstract

The swamp eel ( *Monopterus albus* ), known for its benthic and nocturnal feeding habits, exhibits a keen sense of smell. To date, the genomic basis for its adaptive evolution of olfactory system was poorly investigated. In this study, we performed comparative genomic analysis to reveal the evolutionary processes underlying the expansion and diversification of olfactory receptor (OR) genes in the swamp eel. A total of 629 gene families were identified to expand in the swamp eel, with five (*OLFR*, *GNAL*, *PRKG1*, *NCALD*, and *PDE1*) distributed in olfactory transduction (KEGG map04740). GO enrichment analysis of these expanded gene families showed biological processes related to sensory perception of smell and detection of chemical stimulus. Among the 287 positively selected genes (PSGs) in the swamp eel, *LRRK2*, *ANKDD1B*, *APLNR2*, *SPRED1*, *AFAP1L2*, *PLEK2*, and *PREX2* were linked to signal transduction. A total of 318 OR genes (289 functional genes, 10 partial genes, and 19 pseudogenes) were annotated in the swamp eel genome, significantly surpassing other fish species. Clusters of OR genes were detected on chromosome 4, chromosome 8, chromosome 9, and chromosome 12 in the swamp eel, sharing the same transcriptional orientation, which indicated that tandem duplication was a possible mechanism for the OR gene expansion. Phylogenetic analysis revealed the obvious expansion pattern of OR genes in groups δ and ζ for the swamp eel. Conserved motifs were detected in the OR genes, which indicated the similar functions of OR genes. Our findings unveiled the genomic basis for the olfactory evolution in the swamp eel, providing insights for a better understanding of swamp eel's environmental adaptation and contributing to further investigation of olfactory function in fish. This study enriches the genomic research on the olfactory system of benthic nocturnal fishes and provides a useful evolutionary case for understanding olfactory adaptation to ecological niches in fish.

## Graphical abstract

A total of 629 gene families were identified to expand in the swamp eel, with five (OLFR, GNAL, PRKG1, NCALD, and PDE1) distributed in olfactory transduction (KEGG map04740). A total of 318 OR genes (289 functional genes, 10 partial genes, and 19 pseudogenes) were annotated in the swamp eel genome, significantly surpassing other fish species. Our findings unveiled the genomic basis for the olfactory evolution in the swamp eel, enhancing the understanding of the swamp eel's environmental adaptation and contributing to further investigation of olfactory function in fish.

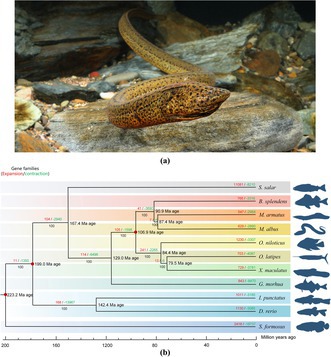

## 1 Background

Olfaction, or the sense of smell, provides important information about the surrounding environment and is essential for animal survival and reproduction, like foraging, social aggregation, mate selection, and predator avoidance (Wang et al. 2015). The information transmitted through a wide array of chemical compounds known as odorants is detected by the olfactory system. The perceiving processes of odorants may vary due to their physical and chemical properties like molecular size, shape, and the presence of specific chemical functional groups (Haddad et al. 2008; Saito et al. 2009). Real‐world odors are often complex mixtures containing tens or hundreds of different odorants, thus the olfactory system must distinguish between these intricate mixtures. The olfactory system's role is to process a complex mix of odorants, produce a response that represents the mixture's composition, and transmit this organized information to the brain (Monahan and Lomvardas 2015). To adapt to various environments and ecological niches, animals have evolved diverse olfactory systems, which could be reflected by the morphological characteristics as well as the composition and regulation of olfaction‐related genes.

Various odor molecules in the environment can be detected by olfactory receptors (ORs), which are expressed in the olfactory epithelium of the nasal cavity (Niimura et al. 2018). ORs, encoded by OR genes, belong to the superfamily of rhodopsin‐like G‐protein‐coupled receptors (GPCRs) with seven‐transmembrane domains and account for the vast discriminatory power of the olfactory system. Vertebrate and invertebrate OR genes have different evolutionary origins (Niimura 2009a, 2009b), and there are significant differences in the OR gene family repertoire owing to their independent evolution for multiple times (Niimura 2012). In vertebrates, ORs exhibit remarkable variation among species and lineages; for example, there is a single intact OR gene in elephant sharks while ~1900 intact genes in elephants (Khan et al. 2015). As a result, the dynamic evolution of the OR repertoire likely aids in adaptation to various ecological niches, such as feeding ecology and habitat (Liu et al. 2021).

In vertebrates, OR genes are categorized into two major types: type I (α, β, γ, δ, ε, and ζ) and type II (η, θ, κ, and λ). It is notable that the groups θ, κ, and λ are likely non‐OR genes, as they have not been found to be expressed in olfactory tissues (Lv et al. 2019). The majority of mammalian OR genes, known as “mammalian‐like” genes, belong to subfamilies α and λ, which are expressed in the air‐filled medial diverticulum and could detect airborne odorants. Subfamilies δ, ε, ζ, and η were identified in fish and termed “fish‐like” genes, which are expressed in the water‐filled lateral diverticulum and could detect water‐soluble odorants. These water‐soluble odorants primarily contain amino acids, prostaglandins, gonadal steroids, and bile acids, which are nonvolatile (Cong et al. 2019). Subfamily β OR genes are found in both aquatic and terrestrial vertebrates, indicating their role in detecting both water‐soluble and airborne odorants (Niimura 2009a, 2009b).

The swamp eel ( *Monopterus albus* ), an important freshwater aquaculture species in China, is economically valuable in virtue of commercial importance and delicious meat (Lv et al. 2022). The fish, commonly found in rice fields, muddy ponds, and swamp areas, is a eurythermal and carnivorous freshwater fish with an exceptional ability to obtain oxygen from the air rather than from water (Pedersen et al. 2014). The swamp eel with benthic and nocturnal feeding habits is known for its burrowing behavior and sensitive sense of smell (Wu et al. 2023). To date, there was no survey on the genetic factors that mediated olfactory responses in the swamp eel at the molecular level. In this study, we conducted comparative genomics analysis to investigate the genetic mechanism underlying the olfactory responses and comprehensively analyzed the nearly complete OR repertoire in the swamp eel. This study could enhance our understanding of the adaptive evolution of olfaction in the fish.

## 2 Materials and Methods

### 2.1 Genome Data Collection

The genome data of the swamp eel was obtained from previous study (Tian et al. 2022). Another 10 fish species (zig‐zag eel [ *Mastacembelus armatus* ; GCF_900324485.2], Siamese fighting fish [ *Betta splendens* ; GCF_900634795.4], Nile tilapia [ *Oreochromis niloticus* ; GCF_001858045.2], Japanese medaka [ *Oryzias latipes* ; GCF_002234675.1], southern platyfish [ *Xiphophorus maculatus* ; GCF_002775205.1], Atlantic cod [ *Gadus morhua* ; GCF_902167405.1], Atlantic salmon [ *Salmo salar* ; GCF_905237065.1], channel catfish [ *Ictalurus punctatus* ; GCF_001660625.3], zebrafish [ *Danio rerio* ; GCF_000002035.6], and Asian arowana [ *Scleropages formosus* ; GCF_900964775.1]) were selected for comparative genomics analyses. All these genome assemblies are at the chromosome level with high quality and integrity, which ensures the reliability and accuracy of subsequent comparative genomic analyses (Liu et al. 2021). Their genome data were downloaded from NCBI (https://www.ncbi.nlm.nih.gov/).

### 2.2 Gene Family Analysis

The orthogroups (OGs) of protein‐coding genes (PCGs) from above 11 fish species were identified using Orthofinder (Emms and Kelly 2019). Whole‐genome all‐vs‐all BLASTP searches were performed with the BLOSUM62 substitution matrix, and homologous sequences were screened with *e*‐value ≤ 1e‐5 and sequence identity ≥ 30% (Altschul et al. 1990; Emms and Kelly 2015). After preliminary clustering using the MCL algorithm with an inflation parameter of 1.5, gene tree construction and clade evaluation were performed using the built‐in pipeline of OrthoFinder. Only monophyletic clades with bootstrap support ≥ 70% and consistent with the species tree topology were defined as final orthogroups (Hillis and Bull 1993; Emms and Kelly 2019). One‐to‐one orthologous genes were aligned using MAFFT (Katoh and Toh 2008) with default settings before phylogenetic tree construction. The ML tree was constructed via FastTree 2 (Price et al. 2010), and the divergence times between species were estimated using MCMCTREE from the PAML package (Yang 2007). Calibration times were retrieved from the TimeTree database (https://www.timetree.org): (i) Asian arowana and zebrafish (215.0–297.9 Mya), (ii) zebrafish and Nile tilapia (180.0–251.5 Mya), and (iii) Nile tilapia and Siamese fighting fish (93.9–110.0 Mya). Gene family expansion was assessed using CAFE (Mendes et al. 2020), with conditional *p*‐values calculated for each gene family. Gene families significantly expanded (*p*‐value below 0.05) were further analyzed functionally using the clusterprofiler R package (Yu et al. 2012).

### 2.3 Positive Selection Analysis

MAFFT (Katoh and Toh 2008) was employed to align the protein sequences for each one‐to‐one orthologous gene from 11 fish species. The multiple protein alignments and corresponding coding sequences were converted into codon alignments using PAL2NAL (Suyama et al. 2006). Positively selected genes (PSGs) in the swamp eel were then identified using the CodeML program in PAML (Yang 2007) with the branch‐site model. A likelihood ratio test was conducted between models, and *p*‐values were obtained by calculating twice the difference in likelihood from the models using the Chi‐squared test. These *p*‐values were further adjusted for multiple testing using the false discovery rate (FDR) method.

### 2.4 Annotation of OR Genes

The above 11 fish species were chosen for OR gene identification and comparison. Initially, known fish OR gene sequences were collected from NCBI using the keyword “olfactory receptor” and manually verified. These sequences were then used as query sequences in a TBALSTN search against the 11 fish genomes (*e*‐value ≤ 1e‐10) (Zhou et al. 2022). The best result, featuring the lowest e‐value and the longest transcript for each gene, was selected for further analysis. All candidate OR genes were verified against the NCBI non‐redundant database (BLASTX) to ensure that the coding sequences matched our search criteria. Domain analysis and annotation of the candidate OR protein set was performed using the hmmscan program from the HMMER3 software package (http://hmmer.janelia.org). Additionally, the transmembrane domains were rechecked using TMHMM‐2.0 (https://services.healthtech.dtu.dk/service.php?TMHMM‐2.0) for the identified OR genes. Finally, the identified OR genes were systematically classified into (1) functional genes: no interrupting stop codons, no frameshifts within the open reading frames (ORFs), and more than 250 amino acids; (2) pseudogenes: early termination, frameshifts within the ORFs, or premature stop codons; and (3) partial genes: no start and/or stop codons or frameshifts within the ORFs (Wang et al. 2021).

### 2.5 Analyses of Chromosomal Distribution, Protein‐Conserved Motif, and Phylogeny

To better visualize the organization of OR genes, the locations of OR genes in the swamp eel were mapped onto chromosomes via TBtools (Chen et al. 2018). Additionally, to understand the evolutionary dynamics of OR genes, conserved motif analysis was performed through Multiple Expectation Maximization for Motif Elicitation (MEME), which could identify novel motifs in unaligned nucleotide or protein sequences (Bailey and Elkan 1994). Each motif was between 5 and 50 amino acids in length, and only the top five conserved motifs were retained. Furthermore, potential N‐glycosylation sites of functional OR genes were predicted using the NetNGlyc server (Gupta et al. 1999). To classify the OR genes of the swamp eel, we conducted phylogenetic analysis based on the known OR genes of the spotted gar ( *Lepisosteus oculatus* ), zebrafish, tongue sole ( *Cynoglossus semilaevis* ), stickleback ( *Gasterosteus aculeatus* ), fugu ( *Takifugu rubripes* ), and European seabass ( *Dicentrarchus labrax* ) together with OR genes of the swamp eel. The translated amino acid sequences of all OR genes were aligned via MAFFT 7 with default parameters, and the ML tree was constructed using FastTree2 (Price et al. 2010) with bootstraps of 1000.

## 3 Result

### 3.1 Gene Family Expansion

**FIGURE 1:**
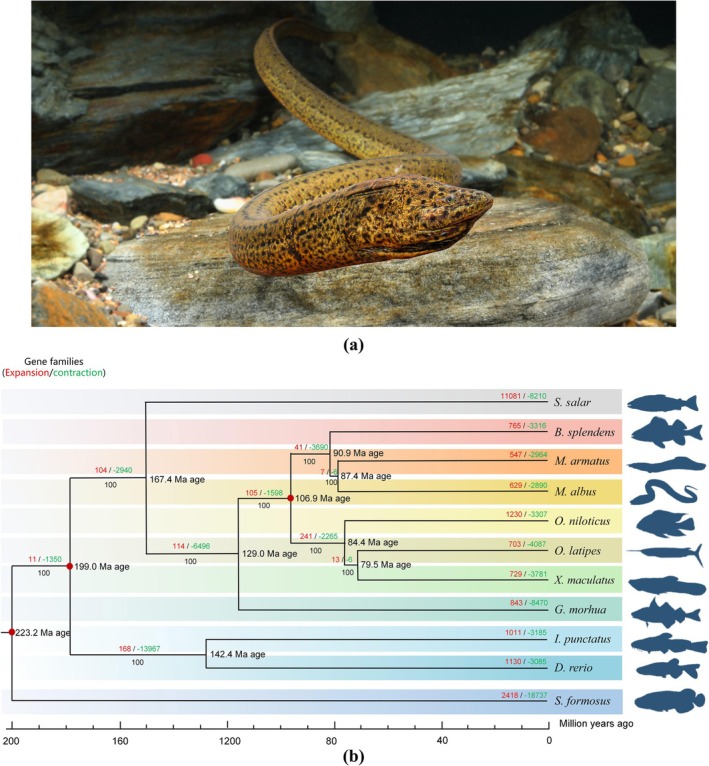
Gene family, phylogeny, and divergence of the swamp eel (a) The Asian swamp eel ( *Monopterus albus* ), the focal species of this study. (b) Time‐calibrated phylogenetic tree of 12 ray‐finned fish species, inferred from single‐copy orthologs. Divergence times were estimated using fossil calibrations. Red/green numbers on branches indicate gene family expansion/contraction (count/total affected genes). Bootstrap support = 100 for all nodes.

**FIGURE 2:**
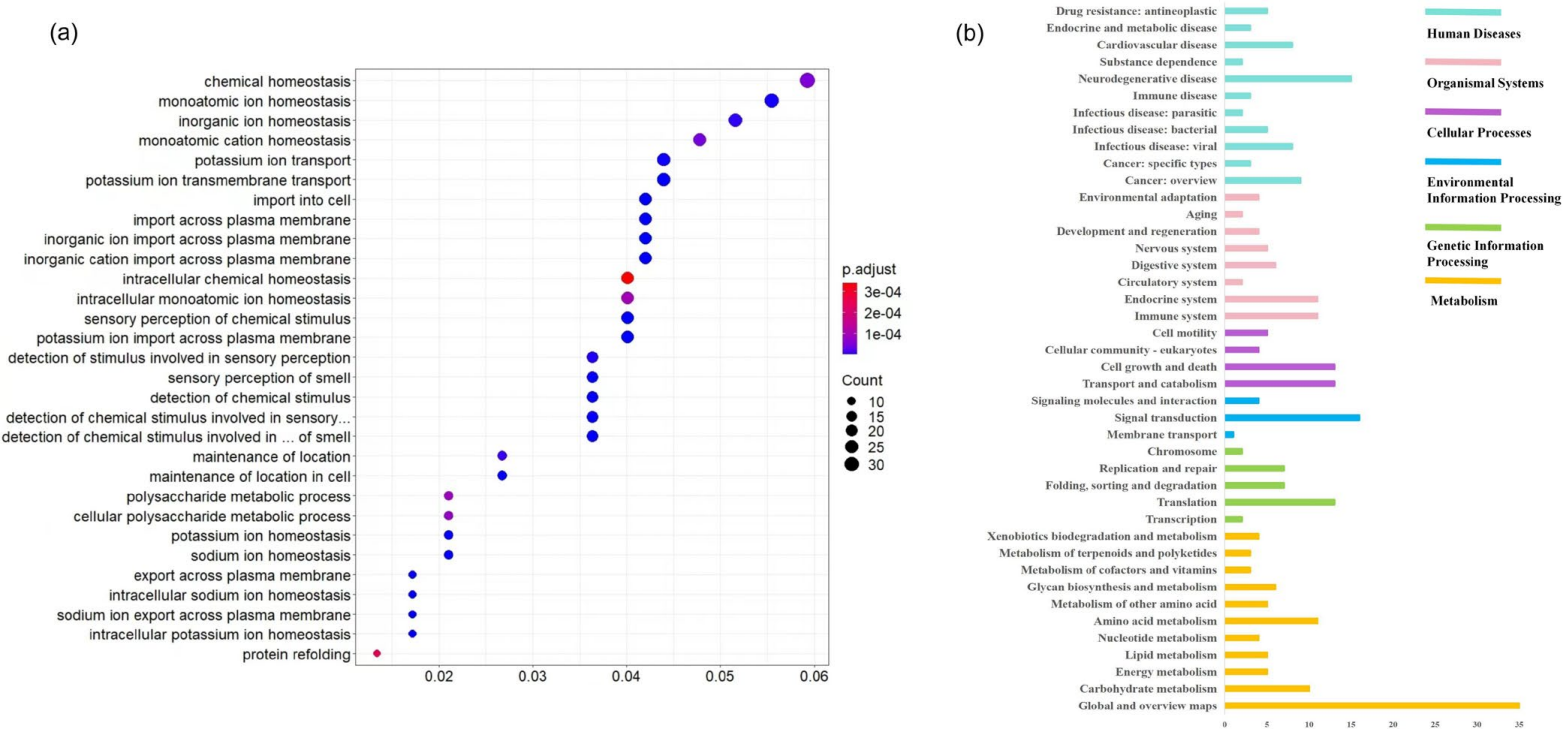
Functional enrichment of expanded gene families and pathway distribution of positively selected genes in the swamp eel. (a) GO enrichment analysis of expanded gene families. (b) KEGG pathway classification of positively selected genes.

**FIGURE 3:**
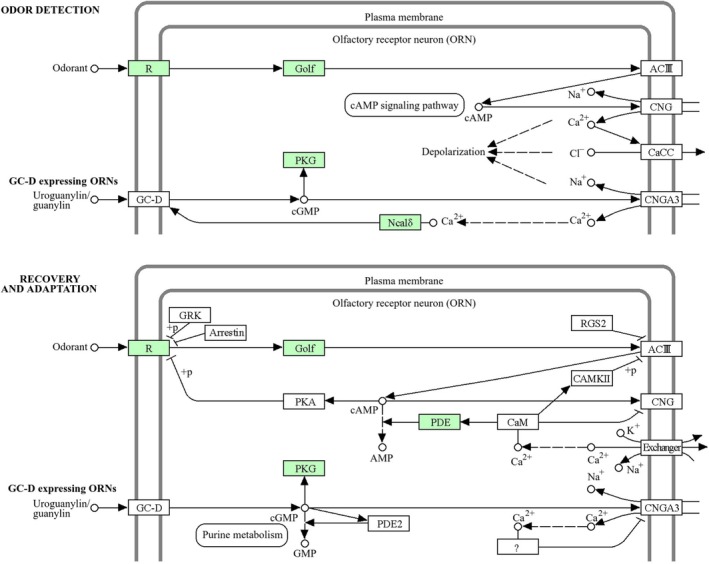
Expanded gene families of the swamp eel distributed in olfactory transduction (KEGG map04740). The 629 expanded gene families of the swamp eel were mapped to KEGG olfactory transduction pathway (map04740). Green highlighted boxes indicate the five significantly expanded gene families (OLFR, GNAL, PRKG1, NCALD, PDE1) in this pathway; gray background is the vertebrate olfactory signal transduction cascade, and arrows show signal transmission direction.

There were 34,783 OGs identified among 11 fish species, and 22,145 PCGs of *M. albus* were detected in 17,468 OGs. Phylogenetic analysis based on 2344 one‐to‐one orthologous genes showed the sister relationship between *M. albus* and *M. armatus* , followed by *B. splendens* (Figure 1). Divergence time analysis showed that *M. albus* diverged from *M. armatus* about 87.4 Mya. A total of 629 gene families were revealed to expand in *M. albus* , which included 2353 PCGs. GO enrichment of these expanded gene families revealed biological processes associated with detection of chemical stimulus involved in sensory perception of smell (GO:0050911, adjusted *p*‐value = 1.39e‐07), sensory perception of chemical stimulus (GO:0007606, adjusted *p*‐value = 1.49e‐07), detection of chemical stimulus involved in sensory perception (GO:0050907, adjusted *p*‐value = 1.49e‐07), detection of chemical stimulus (GO:0009593, adjusted *p*‐value = 1.96e‐07), sensory perception of smell (GO:0007608, adjusted *p*‐value = 3.96e‐07) (Figure 2a). Furthermore, five gene families (*OLFR*, *GNAL*, *PRKG1*, *NCALD*, and *PDE1*) were distributed in olfactory transduction (KEGG map04740) (Figure 3).

### 3.2 Positive Selection

A total of 287 one‐to‐one orthologous genes were identified to have been positively selected in the swamp eel. Based on KEGG annotation analysis, these PSGs were found to distribute in 42 pathways related to metabolism (91 genes), genetic information processing (31 genes), environmental information processing (21 genes), cellular processes (35 genes), organismal systems (45 genes), and human diseases (63 genes) (Figure 2b). Functional analysis based on GO detected three PSGs (*LRRK2*, *ANKDD1B*, and *APLNR2*), two PSGs (*SPRED1* and *AFAP1L2*), and two PSGs (*PLEK2* and *PREX2*) distributed in signal transduction (GO:0006281), regulation of signal transduction (GO:0007165), and intracellular signal transduction (GO:0035556), respectively.

### 3.3 Genomic Organization of OR Gene Repertoires

**FIGURE 4:**
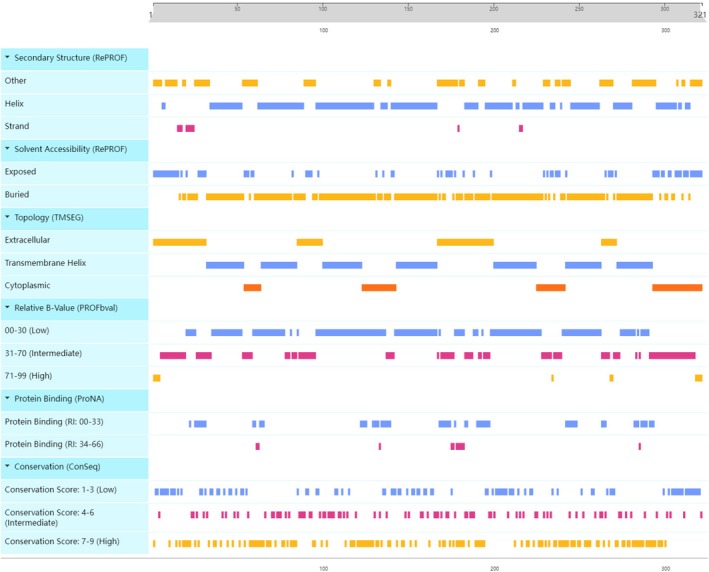
Structure information of ORs of the swamp eel. The *x*‐axis represents amino acid positions (1–321). Six bioinformatic analyses are integrated: secondary structure (RePROF), solvent accessibility (RePROF), transmembrane topology (TMSEG), relative B‐value (PROFbval), protein binding potential (ProNA), and evolutionary conservation (ConSeq). Colored blocks indicate predicted feature types for each residue, and discontinuities reflect transitions between features at adjacent positions.

**FIGURE 5:**
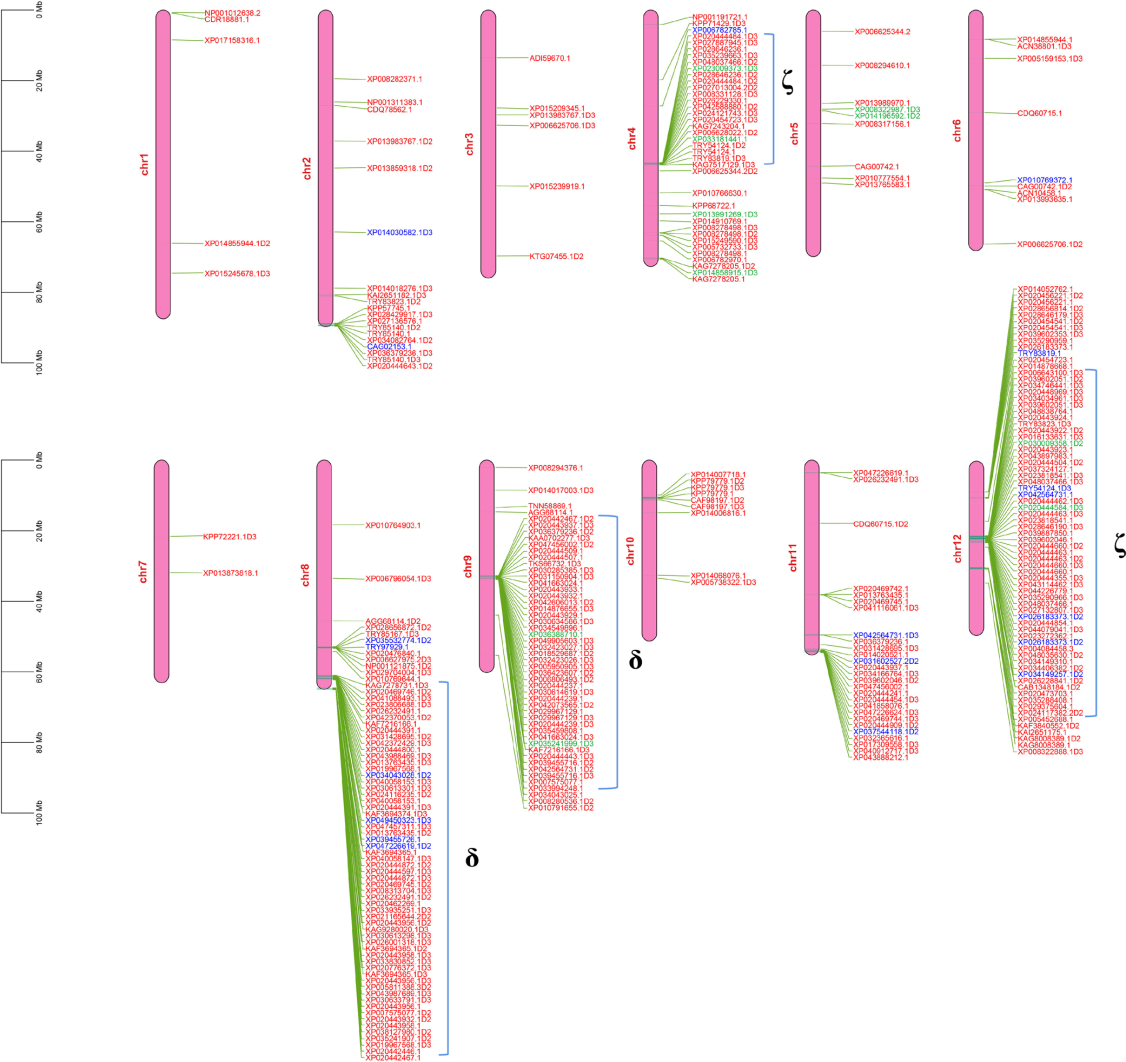
Chromosomal distribution of the OR genes of the swamp eel, with red indicating functional genes, blue denoting pseudogenes, and green representing partial genes. Only the chromosomes with OR gene distribution were shown.

Based on the known OR genes from NCBI as queries, a total of 318 OR genes were identified in the swamp eel, which consisted of 289 functional genes, 10 partial genes, and 19 pseudogenes (Table 1). Structural information such as secondary structure, solvent accessibility, transmembrane helices, and conservation was analyzed for OR genes through PredictProtein (https://predictprotein.org/) (Figure 4). A cluster was defined as a group of OR genes within < 1 Mb of each other, and clusters of OR genes were detected on chromosomes in the swamp eel (Figure 5), which was consistent with previous studies (Zhou et al. 2022, 2023). Typically, OR genes in one cluster on chromosomes like chromosome 4, chromosome 8, chromosome 9, and chromosome 12 shared the same transcriptional orientation, suggesting tandem duplication as a possible mechanism for gene expansion. Further statistical analysis of the OR gene family composition of the major clusters on these four chromosomes revealed obvious family‐specific distribution characteristics: the main clusters on chromosome 4 were composed entirely of ζ family OR genes, those on chromosomes 8 and 9 were all δ family OR genes, and the major clusters on chromosome 12 consisted exclusively of ζ family OR genes. This distinct distribution pattern further confirmed the high family specificity of tandem duplication events of OR genes in the swamp eel genome. The swamp eel had the largest number of OR genes (318) among the 11 fish species studied, which was over three times as many as the Japanese medaka (Table 1). There were 289 functional OR genes in the swamp eel, which also significantly exceeded other fish species. The proportion of partial OR genes was very low in each species, ranging from 0.56% to 4.80%. In the swamp eel, 318 OR genes were mapped to chromosomes, with chromosome 8 having the most OR genes (*n* = 71) and functional OR genes (*n* = 65) (Table 2). There were only two OR genes distributed on chromosome 7, which was the least among all 12 chromosomes. According to the OR gene distribution on chromosomes, it seemed there was no correlation between the OR gene number and chromosome length.

**TABLE 1:** Summary of the olfactory receptor (OR) genes in 11 fish species based on chromosome‐level genomes.

Species	No. of functional OR genes (%)	No. of partial OR genes (%)	No. of OR pseudogenes (%)	Total number of OR genes
*Monopterus albus*	289 (90.88)	10 (3.14)	19 (5.97)	318
*Mastacembelus armatus*	192 (93.66)	2 (0.98)	11 (5.37)	205
*Betta splendens*	114 (96.61)	4 (3.39)	0 (0)	118
*Oreochromis niloticus*	144 (91.72)	4 (2.55)	9 (5.73)	157
*Oryzias latipes*	82 (95.35)	1 (1.16)	3 (3.49)	86
*Xiphophorus maculatus*	99 (94.29)	1 (0.95)	5 (4.76)	105
*Gadus morhua*	87 (95.60)	1 (1.10)	3 (3.30)	91
*Salmo salar*	164 (92.66)	1 (0.56)	12 (6.78)	177
*Ictalurus punctatus*	89 (94.68)	2 (2.13)	3 (3.19)	94
*Danio rerio*	158 (91.86)	1 (0.58)	13 (7.56)	172
*Scleropages formosus*	119 (95.20)	6 (4.80)	0 (0)	125

**TABLE 2:** Composition of OR genes for each chromosome of the swamp eel.

Location	Chromosome size (Mb)	No. of functional OR genes	No. of partial OR genes	No. of OR pseudogenes	Total number of OR genes
Chr1	87.97	5	0	0	5
Chr 2	90.20	17	0	2	19
Chr 3	76.37	6	0	0	6
Chr 4	73.15	33	4	1	38
Chr 5	70.33	7	2	0	9
Chr 6	68.68	8	0	1	9
Chr 7	63.48	2	0	0	2
Chr 8	65.39	65	0	6	71
Chr 9	60.65	48	2	0	50
Chr 10	51.71	9	0	0	9
Chr 11	55.67	24	0	3	27
Chr 12	49.80	65	2	6	73

### 3.4 Conserved Motif and Classification

**FIGURE 6:**
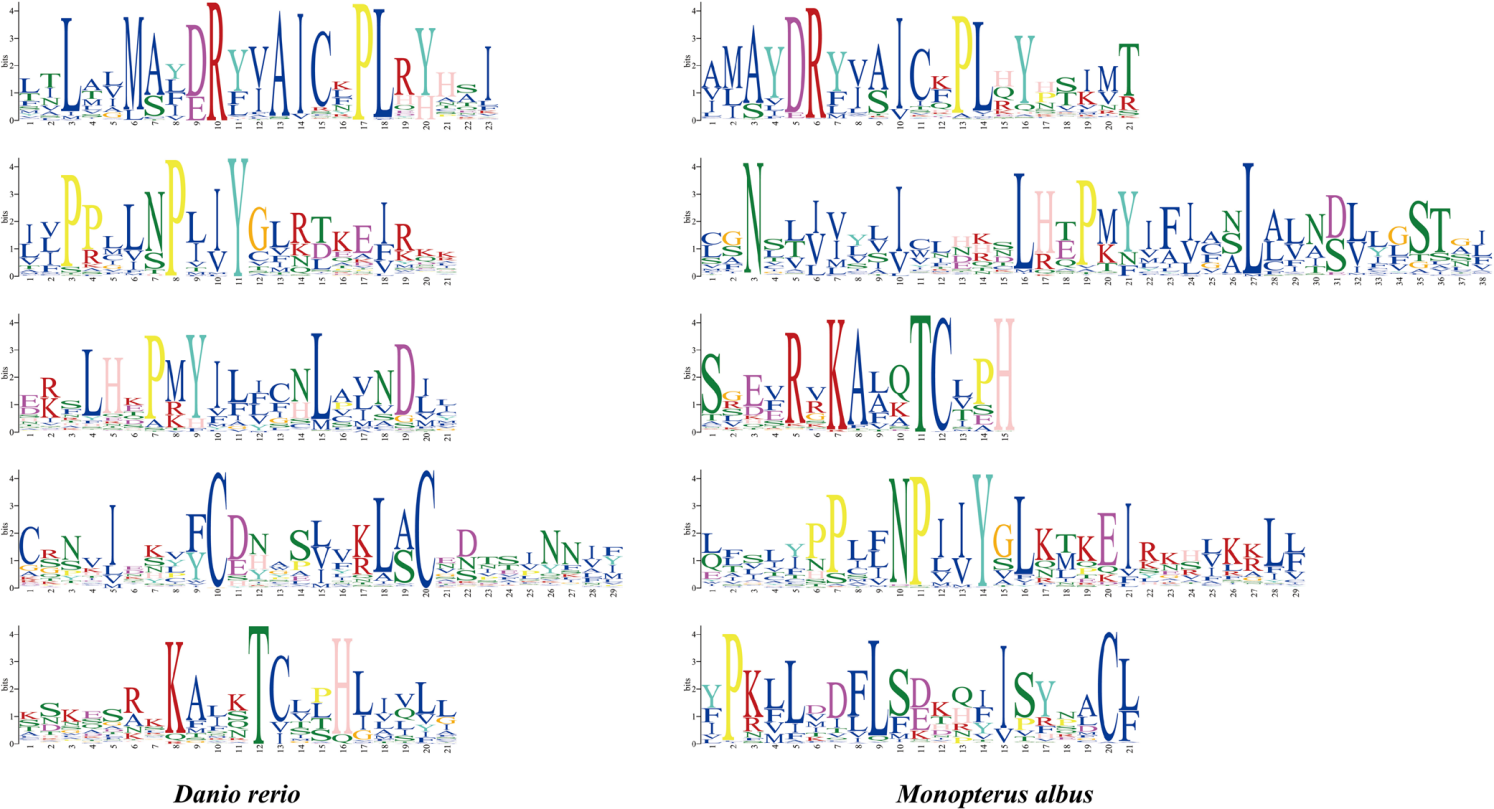
Logo representation of the five best conserved motifs identified for OR genes of the zebrafish and swamp eel.

**FIGURE 7:**
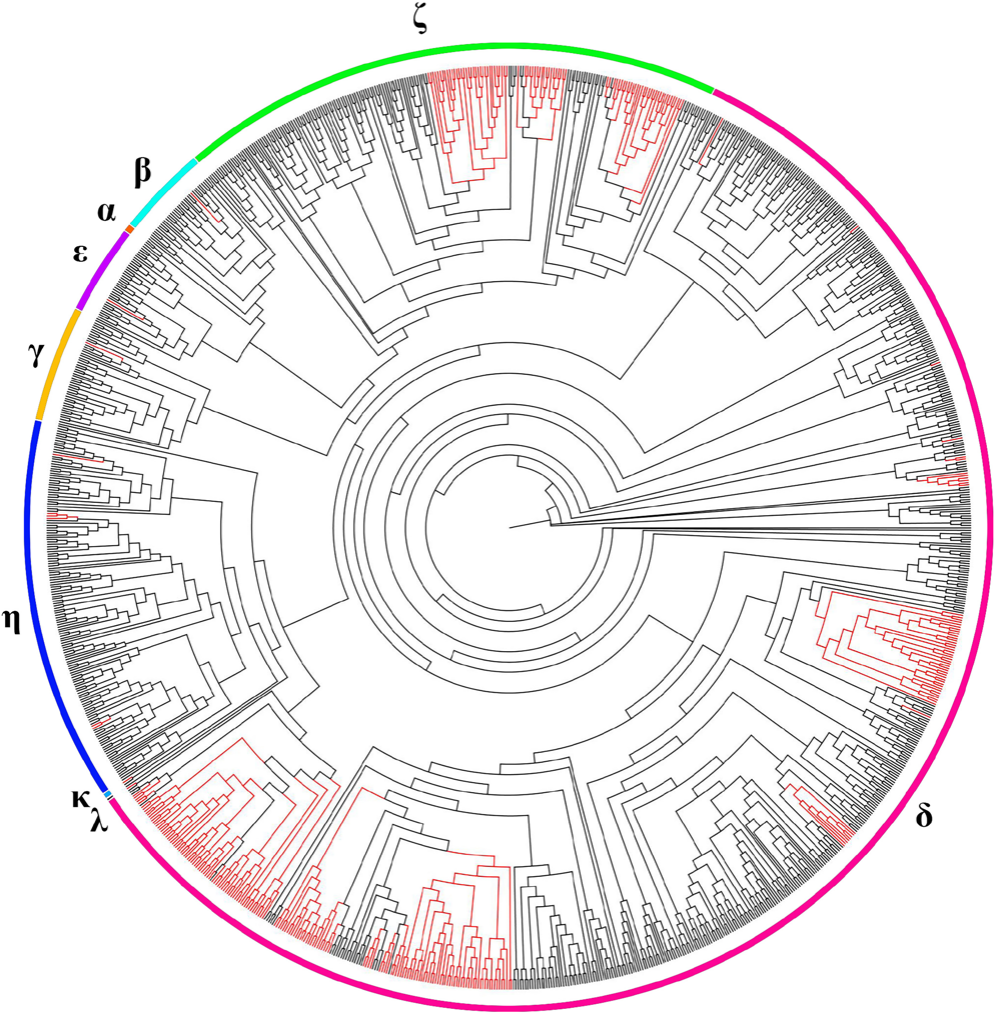
The phylogenetic tree of OR genes from six fish species (swamp eel, spotted gar, zebrafish, tongue sole, stickleback, fugu, and European seabass). The swamp eel was represented by red color.

Conserved amino acids are typically found in homologs of a single gene but can also occur in all homologs of gene pairs. To identify conserved amino acids in OR genes of the swamp eel, we performed the motif search and found highly conserved motifs such as “MADRYAIC”. The frequency of each amino acid was represented by its height. Figure 6 illustrated the top five most conserved motifs in the OR genes of the swamp eel and zebrafish. Although these conserved motifs were not identical between the swamp eel and zebrafish, the motifs shared similar sequence composition, respectively. OR genes with the conserved motifs exhibited similar functions at the protein level across different species. Additionally, the N‐glycosylation sites were detected in most functional OR genes. According to the phylogenetic tree, the OR genes were clearly classified into two clades (type I and type II), and seven groups were identified in the swamp eel OR repertoire, including type I (β, γ, δ, ε, and ζ) and type II (η and κ) (Figure 7). Moreover, an obvious expansion pattern of swamp eel OR genes was revealed in groups δ and ζ.

## 4 Discussion

### 4.1 OR Function and Identification

Olfaction is a key chemosensory process forming the sense of smell, crucial for the survival and reproduction of animals. Fish have developed highly sophisticated olfactory systems to detect, distinguish, and interpret a wide array of water‐soluble chemical signals which provide critical information on food sources, danger, kin recognition, and spawning migration. Fish olfactory perception begins with specific interactions between odorants and ORs in sensory neurons within the olfactory epithelium. These interactions create distinct odorant maps in the olfactory bulb, which are then transmitted to higher olfactory centers in the brain, triggering various physiological and behavioral responses (Miyasaka et al. 2014). Previous research of the OR subgenomes of vertebrates has uncovered substantial variations in OR gene composition, indicating that the olfactory systems of vertebrates are heavily influenced by natural selection (Lee et al. 2013). Investigating these genetic variations in ORs can provide insights into animal evolution in response to environmental changes.

In this study, we conducted comparative genomics analysis and characterized the OR gene repertoire of the swamp eel based on the chromosome‐level genome. We identified 318 OR genes in the swamp eel, which was significantly higher than the other 10 fish species, ranging from 86 to 205 OR genes. Furthermore, the number of functional OR genes in the swamp eel was also the largest, which was about 3.5 times as large as that in *Oryzias latipes* . These differences in OR gene repertoires revealed significant variation in the genetic component of OR systems among fish. It should be noted that the results of OR identification in this study have some differences compared to previous studies (Wang et al. 2021; Zhu et al. 2017). The unexpected discrepancies in the estimated number of OR genes identified across different fish species may be due to the quality of the genomes and/or the varying bioinformatics search strategies employed. The use of varied annotation strategies across different organisms complicates the comparison of OR genes between species; thus, OR comparison between different fish species is relatively more credible. Moreover, the rapid development of sequencing technologies could improve the quality of genome assembly, which in turn enhances the accuracy of OR gene identification. In sum, a standardized and uniform approach to annotate OR gene repertoires across various fish species should be developed. Additionally, it remains to be verified whether the number of OR genes in the swamp eel was larger than that of other fish species.

### 4.2 Adaptive Evolution of Olfaction in the Swamp Eel

Most fish have a highly developed olfactory system with relatively large olfactory bulbs (OB) and olfactory epithelium (OE). However, the number of OR genes varies significantly among fish species. A cross‐species analysis of the 11 studied fish confirmed no significant positive correlation between OR gene number and genome size. Notably, the swamp eel possessed the largest number of OR genes among all examined species, whereas its genome size was much smaller than that of species such as Atlantic salmon ( *Salmo salar* ) and zebrafish ( *Danio rerio* ). Moreover, species with similar genome sizes (e.g., Japanese medaka and southern platyfish, Nile tilapia and Siamese fighting fish) showed distinctly different OR gene counts, further indicating that OR gene family expansion or contraction is independent of genome size. Meanwhile, the chromosomal distribution of OR genes in the swamp eel was also uncoupled from chromosome size: OR genes were highly enriched in several medium‐ and small‐sized chromosomes (especially Chr8 and Chr12) rather than in the largest chromosomes. This uneven distribution mainly resulted from tandem duplication events that formed dense OR clusters, rather than chromosome length itself. These results clearly rule out genome size and chromosome size as major drivers of OR gene variation in fishes. Instead, they provide solid empirical evidence that OR gene repertoire diversification is primarily shaped by species‐specific habitat and life‐history traits. We hypothesize that this variation in OR genes is likely influenced by the different habitats and lifestyles of the fish. The presence of unique or common OR genes among different species indicates the diversification or retention of orthologous genes from shared ancestors during species evolution (Lee et al. 2013). Through comparison of the structural characteristics of OR genes, previous studies have indicated that the evolutionary increase in the number of OR genes could be driven by the increase in gene numbers within subfamilies and the increase in the number of subfamilies (Zhou et al. 2022, 2023). In this study, compared to the other 10 fish species with OR gene number ranged from 86 to 205, the swamp eel had the largest number of OR genes (*n* = 318). This suggests that the OR gene family has expanded during the evolution of the swamp eel. Specifically, OR gene expansion was detected in subfamilies δ and ζ. The expanded OR gene families seem to enhance the swamp eel's sensitivity to specific olfactory cues that are prevalent or essential in its chemical environment. The swamp eel possesses only 19 pseudogenes among 318 OR genes, representing a low pseudogene proportion. This is presumably attributed to strong purifying selection acting on its olfactory system, which effectively inhibits the pseudogenization of OR genes (Nei et al. 2008). As a benthic, nocturnal, burrowing fish with degenerated vision, it relies heavily on olfaction for survival and reproduction. Natural selection therefore preserves functional OR gene copies while eliminating mutated sequences prone to pseudogenization (Zeng et al. 2012; Bear et al. 2016). This evolutionary pattern is consistent with that of the blunt snout bream ( *Megalobrama amblycephala* ) (Liu et al. 2021). OR genes with over 60% sequence homology have been reported to bind to odorants with similar chemical structures, thus orthologous OR genes within the same subfamily across different species might detect similar odorant substances (Zhou et al. 2022). Supporting this possibility, distinct subfamilies within OR gene families were found to evolve independently, likely as a response to various chemical cues that were associated with each species' specific ecological niche. Previous studies conducted on reptiles, birds, and mammals have indicated that the expansion and contraction of OR genes are linked to significant shifts in habitat and lifestyle (Liu et al. 2021). For instance, investigation into OR diversity across two reptiles and 48 birds revealed species and lineage‐specific variations in OR gene subfamilies related to adaptations to aquatic and terrestrial environments (Vandewege et al. 2016). Similarly, investigation of OR genes in sauropsida showed that adaptation associated with the evolution of life history have influenced the unique OR repertoires observed in different members of this subfamily (Khan et al. 2015).

In animal evolution, tandem gene duplication is considered to be the primary source of new genes (Yang et al. 2005), and the functions of these new genes, influenced by genetic drift, may be related to environmental adaptation or could be neutral (Pedersen et al. 2014). Notably, OR genes generated by tandem duplication frequently cluster into phylogenetically distinct subfamilies, a phenomenon attributed to neofunctionalization during adaptive evolution (Innan and Kondrashov 2010). After duplication, redundant OR gene copies accumulate adaptive mutations in ligand‐binding domains, acquiring novel odorant recognition properties that enable detection of distinct environmental odorants. This functional divergence provides a reasonable explanation for the formation of diverse OR subfamilies from tandem duplication (Kondo et al. 2002; Beisswanger and Stephan 2008). In addition to tandem duplication, retrotransposition is another important evolutionary mechanism that is likely to contribute to the expansion of the OR gene family in the swamp eel: the retrotransposition of parental OR genes generates new single‐exon OR gene copies, which can be further fixed and amplified in the genome under natural selection, thus enriching the diversity of the OR gene repertoire. While gene copy number variations resulting from duplication are often deemed neutral, they can be selectively employed and maintained in the genome for adaptation to new environments or habitats owing to specific selection pressures (Nei et al. 2008; Ramdya and Benton 2010). This phenomenon has been observed in teleosts (Korsching 2009), where certain OR genes have undergone species‐specific expansion through further gene duplication, potentially aiding in adaptive evolution. Owing to the degeneration of the eye, the swamp eel as a burrowing animal mainly depends on their sensitive sense of smell to find food (Zeng et al. 2012). The substantial expansion of OR genes in the swamp eel genome observed in the present study possibly compensated its eye degeneration to some extent. The distinct evolutionary patterns in the swamp eel strongly suggest that the birth‐and‐death process and relaxed selection have resized and reshaped the OR gene families (Bear et al. 2016). Further investigation is essential to reveal the information about the molecules recognized by OR individuals and how the swamp eel responds to these odorants. Furthermore, seven PSGs (*LRRK2*, *ANKDD1B*, *APLNR2*, *SPRED1*, *AFAP1L2*, *PLEK2* and *PREX2*) possibly help enhance the olfactory ability of the swamp eel. For example, olfactory dysfunction has been observed in Parkinson's disease (PD) patients carrying the LRRK2 G2019S variant in Caucasians (Cao et al. 2016), which indicates that LRRK2 plays a pivotal role in olfactory system. Besides OR, *GNAL*, *PRKG1*, *NCALD*, and *PDE1* gene families distributed in olfactory transduction (KEGG map04740) were also found to expand in the swamp eel, which was likely to enhance the olfactory ability. Previous study has confirmed that *GNAL* is expressed in brain outside the areas responsible for olfaction (Vuoristo et al. 2000). *PDE1* was reported to be highly localized in olfactory cilia (Moon et al. 2005).

## 5 Conclusions

In summary, we performed comparative genomics analysis to investigate the adaptive evolution of the olfactory system in the swamp eel. We detected 629 gene families expanded in the swamp eel, of which five gene families (*OLFR*, *GNAL*, *PRKG1*, *NCALD*, and *PDE1*) were distributed in olfactory transduction (KEGG map04740). Among 287 PSGs, *LRRK2*, *ANKDD1B*, *APLNR2*, *SPRED1*, *AFAP1L2*, *PLEK2*, and *PREX2* were associated with signal transduction. A total of 318 OR genes, including 289 functional genes, were detected in the swamp eel, which was significantly higher than other fish species. What is more, we analyzed chromosomal distribution, classification, and conservative motifs of OR genes. Our results revealed the genomic basis of adaptive evolution of the olfactory system in the swamp eel, which would help better understand its environmental adaptation and lay a solid foundation for further study of OR gene evolution in fish. It also enriched the molecular evidence for the co‐evolution of fish olfactory genes and ecological habits and offered a genomic reference for teleost olfactory evolution research.

## Author Contributions

**Chuang Zhou:** formal analysis (equal), writing – original draft (equal). **Zhongyi Wang:** formal analysis (equal). **Chenhao Zhang:** formal analysis (equal). **Zhaobin Song:** supervision (equal), writing – review and editing (equal).

## Funding

This work was supported by National Natural Science Foundation of China (32501374).

## Conflicts of Interest

The authors declare no conflicts of interest.

## Data Availability

The genome data of the swamp eel was obtained from previous study (Tian et al. 2022). Another 10 fish species (zig‐zag eel [ *Mastacembelus armatus* ; GCF_900324485.2], Siamese fighting fish [ *Betta splendens* ; GCF_900634795.4], Nile tilapia [ *Oreochromis niloticus* ; GCF_001858045.2], Japanese medaka [ *Oryzias latipes* ; GCF_002234675.1], southern platyfish [ *Xiphophorus maculatus* ; GCF_002775205.1], Atlantic cod [ *Gadus morhua* ; GCF_902167405.1], Atlantic salmon [ *Salmo salar* ; GCF_905237065.1], channel catfish [ *Ictalurus punctatus* ; GCF_001660625.3], zebrafish [ *Danio rerio* ; GCF_000002035.6], and Asian arowana [ *Scleropages formosus* ; GCF_900964775.1]) were selected for comparative genomics analyses, and their genome data were downloaded from NCBI (https://www.ncbi.nlm.nih.gov/).
